# Update on Perioperative Prevention of Cardiac Surgery-Associated Acute Kidney Injury

**DOI:** 10.3390/jcm15176532

**Published:** 2026-08-24

**Authors:** Luis Baeza, Pablo Avanzas, Carla Delgado-Martí, Manuel García-Delgado, Santiago Gómez-Estanga, José M. López González, Pablo Montero-López, Marc Vives

**Affiliations:** 1 Department of Anesthesiology and Critical Care, Hospital Universitario Central de Asturias, 33011 Oviedo, Spain; luisbaeza93@gmail.com (L.B.); josemalpz@gmail.com (J.M.L.G.); 2 Heart Area, Hospital Universitario Central de Asturias, 33011 Oviedo, Spain; 3 Instituto de Investigación Sanitaria del Principado de Asturias (ISPA), 33011 Oviedo, Spain; 4 Department of Medicine, University of Oviedo, 33003 Oviedo, Spain; 5 Department of Anesthesiology and Critical Care, Hospital Universitario Alvaro Cunqueiro, 36312 Vigo, Spain; carla.delgado.marti@sergas.es; 6 Intensive Care Unit, Hospital Virgen de las Nieves, 18014 Granada, Spain; mjgardel@hotmail.com; 7 Instituto de Investigación Biosanitaria ibs.GRANADA, 18012 Granada, Spain; 8 Intensive Care Unit, Complexo Hospitalario Universitario de A Coruña, 15006 A Coruña, Spain; sgomezestanga@gmail.com; 9 Department of Anesthesiology and Critical Care, Clínica Universidad de Navarra, 31008 Pamplona, Spain; pmonterol@unav.es (P.M.-L.); mvives@unav.es (M.V.); 10 Instituto de Investigación Sanitaria de Navarra (IdiSNA), 31008 Pamplona, Spain

**Keywords:** acute kidney injury, cardiac surgery, cardiac surgery-associated acute kidney injury, cardiopulmonary bypass, goal-directed perfusion, amino acid infusion, renoprotection, KDIGO bundle, renal replacement therapy

## Abstract

Cardiac surgery-associated acute kidney injury (CS-AKI) increases short- and long-term mortality, progression to chronic kidney disease (CKD), and healthcare costs. Its pathogenesis is multifactorial—combining renal hypoperfusion, impaired oxygen delivery, hemodilution, inflammation, ischemia–reperfusion injury, and nephrotoxin exposure—so no single intervention confers universal protection. This narrative review appraises fourteen perioperative prevention strategies, grading each by study design, reproducibility, and concordance with contemporary guidelines. The strongest actionable evidence supports the preservation of renal oxygen delivery during cardiopulmonary bypass through goal-directed perfusion, perioperative amino acid infusion, and biomarker-guided Kidney Disease: Improving Global Outcomes (KDIGO) care bundles. Remote ischemic preconditioning, pulsatile flow, minimally invasive extracorporeal circulation, dexmedetomidine, N-acetylcysteine, levosimendan, hemoadsorption with the oXiris membrane, and natriuretic peptides show variable or subgroup-dependent signals limited by heterogeneous trial design and acute kidney injury (AKI) definitions. Prevention of CS-AKI is, therefore, best conceived as a multimodal, patient-centered process integrating preoperative risk stratification, intraoperative oxygen delivery optimization, patient blood management (PBM), and postoperative nephrotoxin avoidance and surveillance.

## 1. Introduction

Acute kidney injury (AKI) complicates 20–40% of cardiac surgical procedures, with renal replacement therapy (RRT) required in 1.6–5.8% of affected patients [1,2,3]. Perioperative mortality increases by a factor of 3–8 in patients who develop cardiac surgery-associated acute kidney injury (CS-AKI) [2,4], and in the subgroup requiring dialysis, 30-day mortality reaches 64% compared with 4% in patients without renal injury [5]. Long-term sequelae further compound the burden: 5- and 7-year survival rates of 54% and 38%, respectively, have been reported following CS-AKI [2], and approximately 25% of patients progress to chronic kidney disease (CKD) within 3 years of the index event [2]. The economic dimension is proportional: in the United Kingdom, hospital costs attributable to AKI were estimated at £1.02 billion annually—approximately €1.2 billion—representing more than 1% of the total National Health Service (NHS) budget [6], and a systematic review currently registered on PROSPERO [7] is under way to consolidate the global economic evidence base.

The pathogenesis of CS-AKI is multifactorial: renal hypoperfusion during cardiopulmonary bypass (CPB), systemic inflammatory response, ischemia–reperfusion injury, hemolysis, and nephrotoxin exposure act in concert, with their relative contributions varying according to the patient risk profile, surgical complexity, and perfusion strategy [8,9,10]. Risk stratification tools, such as Cleveland Clinic Score [11], Mehta Score [12], and EuroSCORE II [13], allow for preoperative identification of high-risk patients, yet identification alone has not translated into a proportional reduction in incidence [8].

This narrative review systematically appraises fourteen perioperative intervention domains, ranging from perfusion optimization and pharmacological strategies to anesthetic management and extracorporeal circuit design, with evidence graded according to study design, reproducibility across randomized trials, consistency with contemporary guideline recommendations, and clinical feasibility.

## 2. Methods

### 2.1. Literature Search Strategy

This is a narrative review, and it did not follow a systematic search protocol. Data were gathered through a search of electronic databases to identify studies related to intraoperative strategies to prevent CS-AKI published between 2004 and 2026. The search was conducted in PubMed, Google Scholar, Scopus, and Web of Science. The search terms and keywords included cardiac surgery, cardiopulmonary bypass, acute kidney injury, renal protection, goal-directed perfusion, oxygen delivery, pulsatile flow, remote ischemic preconditioning, minimally invasive extracorporeal circulation, mean arterial pressure, hemodynamic management, preoperative anemia, red blood cell transfusion, N-acetylcysteine, amino acid infusion, renal functional reserve, extracorporeal blood purification, hemoadsorption, volatile anesthesia, and total intravenous anesthesia. The lower date limit of 2004 corresponds to the publication of the first consensus definition of acute kidney injury (the Risk, Injury, Failure, Loss of kidney function, and End-stage kidney disease [RIFLE] criteria). This milestone initiated the standardization of diagnostic frameworks across clinical trials, thereby ensuring that the evaluated contemporary prevention strategies are contextualized within consistent and reproducible renal endpoints. All studies included were human studies and published in English. As this was a narrative review, no formal systematic screening process (such as the Preferred Reporting Items for Systematic Reviews and Meta-Analyses [PRISMA]) was applied, and the study selection was based on the relevance to the predefined clinical scenarios.

The landmark studies included in Table 1 were selected according to the following criteria: (1) randomized controlled trials, large multicenter cohort studies, or meta-analyses directly evaluating one of the fourteen prevention strategies against a defined comparator; (2) explicit use of a standardized AKI definition (RIFLE, Acute Kidney Injury Network [AKIN], or Kidney Disease: Improving Global Outcomes [KDIGO]) or, where a landmark trial reported renal events only as a component of a composite outcome or according to study-specific criteria, a clearly defined renal endpoint (e.g., renal failure or renal replacement therapy); in such cases, the study was retained because of its practice-informing value, and the nature of the renal endpoint is specified in Table 1 and taken into account when grading the strength of the evidence; (3) sufficient sample size and/or methodological rigor to be considered as practice-informing (e.g., pivotal trials cited by current international guidelines) or, where large trials were unavailable, the best available evidence for that domain. Additional studies that reported AKI as an outcome in CS-AKI prevention but did not meet the criteria for inclusion as landmark trials are provided in the Supplementary Materials (Table S1).

### 2.2. Scope and Structure

Fourteen intervention domains are addressed in descending order of evidence quality and clinical actionability. Goal-directed perfusion is presented first as the physiological framework within which all remaining strategies operate, followed by amino acid infusion—currently the pharmacological intervention with the strongest contemporary multicenter randomized evidence for reducing creatinine-defined CS-AKI. Preoperative anemia correction is then addressed as a critical, modifiable risk context for optimal patient preparation. The biomarker-guided Kidney Disease: Improving Global Outcomes (KDIGO) care bundle follows, carrying a formal guideline recommendation with a 1B recommendation in the 2026 KDIGO public review draft [53]—and positive multicenter randomized evidence. Remote ischemic preconditioning, pulsatile flow, and minimally invasive extracorporeal circulation are presented as interventions with moderate-quality evidence and inconsistent clinical translation, followed by dexmedetomidine, for which consistent meta-analytic signals have not yet been incorporated into guideline recommendations. Intraoperative pressure targets, volatile anesthetic agents, and intraoperative transfusion strategies (liberal versus restrictive) are then addressed as high-quality evidence of no renal benefit—a category conceptually distinct from the preceding interventions, in that the absence of effect has been demonstrated by adequately powered multicenter randomized trials rather than inferred from underpowered or heterogeneous data. This distinction carries direct practice implications: where moderate-quality positive signals invite further trials, high-quality null results redirect attention away from these targets toward strategies with demonstrable renal effect. N-acetylcysteine and levosimendan are addressed as agents with subgroup-specific signals unconfirmed in large pragmatic trials. Extracorporeal blood purification with the oXiris membrane and natriuretic peptides are addressed last, reflecting preliminary evidence or geographic constraints that have limited clinical translation. Although this was designed as a narrative review rather than a systematic review, evidence was prioritized according to study design, sample size, reproducibility across randomized trials and meta-analyses, consistency with contemporary guideline recommendations, biological plausibility, and clinical feasibility. The landmark studies supporting each of these strategies are summarized in Table 1, which reports for every study the design, population, intervention versus comparator, AKI endpoint, and key CS-AKI result. The consolidated strength of recommendation for each of the fourteen interventions is summarized in Table 2.

When the evidence across studies was discordant, precedence was given to the results from adequately powered multicenter randomized controlled trials and meta-analyses over single-center or underpowered studies, as well as to findings replicated across more than one trial over isolated positive results. Where discordance persisted despite this hierarchy—for example, differing effect estimates depending on the AKI definition applied—this is explicitly noted in the text and in Table 1, and the corresponding domain was categorized as demonstrating “moderate-quality” or “subgroup-specific” evidence rather than assigned a higher-certainty tier.

## 3. Perioperative Strategies

The fourteen domains addressed in Section 3 were selected because each represents a perioperative intervention with a defined causal hypothesis for CS-AKI prevention, dedicated randomized or large observational evidence, and/or explicit mention in a current international clinical practice guideline. Interventions lacking dedicated clinical trial evidence in the CS-AKI setting or falling outside the perioperative window defined for this review were not included as standalone domains.

### 3.1. Goal-Directed Perfusion

The association between oxygen delivery (DO_2_) during CPB and postoperative AKI is biologically plausible given the metabolic vulnerability of the renal medulla, where oxygen tension is naturally low (10–20 mmHg), making it susceptible to ischemia during periods of severe hemodilution or low pump flow [56,57]. Ranucci et al. identified nadir DO_2_ as the major predictor of CS-AKI, with a critical threshold estimated at 272 mL/min/m^2^ [56]. Subsequent data corroborated these findings, establishing a nadir DO_2_ < 262 mL/min/m^2^ as an independent risk factor for AKI stage 2 [14].

High-level evidence for goal-directed perfusion (GDP) was provided by the GIFT trial, a multicenter randomized controlled trial (RCT), which demonstrated that maintaining DO_2_ ≥ 280 mL/min/m^2^ through pump flow adjustments significantly reduced Acute Kidney Injury Network (AKIN) stage 1 AKI (relative risk [RR]: 0.45; 95% confidence interval [CI]: 0.25–0.83) [15]. These results were recently extended by another RCT utilizing a higher threshold of 300 mL/min/m^2^, which reported a significant reduction in overall AKI incidence (14.6% vs. 30.4%) [16]. Subgroup analyses suggest that GDP may be particularly effective in patients with smaller body surface area or lower nadir hematocrit, where hemodilutional effects are most pronounced.

Recent evidence also emphasizes that the duration of low DO_2_ may be more important than the absolute nadir value. The largest area under the curve (AUC) below 300 mL/min/m^2^ (representing the most severe single episode of oxygen debt) was a more accurate predictor of AKI than cumulative AUC or nadir DO_2_ [58]. Guidelines now issue a Class I, Level A recommendation for GDP to reduce early stages of AKI, specifically advising management aimed at limiting the length of CPB time spent with low DO_2_ values [54]. The potential benefit of individualizing DO_2_ targets according to preoperative pulse pressure or real-time cerebral autoregulation monitoring remains unresolved.

Based on current evidence, maintaining a DO_2_ index (DO_2_i) ≥ 280 mL/min/m^2^ during CPB is recommended to reduce the incidence of CS-AKI, given its physiological rationale, safety profile, and consistent supportive data.

### 3.2. Amino Acid Infusion

The kidneys possess the capacity to augment the glomerular filtration rate (GFR) in response to amino acid loads through afferent arteriolar vasodilation, attenuation of tubuloglomerular feedback, and increased cortical production of nitric oxide (NO) and prostaglandins—a phenomenon termed recruitment of renal functional reserve (RFR) [59,60]. In experimental models using non-anesthetized sheep, intravenous amino acid infusion increased GFR and improved both medullary and cortical oxygenation, directly addressing the intraoperative renal hypoxia central to CS-AKI pathogenesis [60]. This mechanistic rationale informed the design of the PROTECTION trial, which randomized 3511 adult cardiac surgery patients to 2 g/kg/day of intravenous amino acids or placebo [17]. Amino acid infusion produced a 15% relative risk reduction in AKI (26.9% vs. 31.7%; *p* = 0.002) and nearly halved the incidence of stage 3 AKI (1.6% vs. 3.0%; RR 0.56). A subsequent meta-analysis of seven randomized controlled trials totaling 5059 patients reported a pooled RR for AKI of 0.81 (95% CI 0.68–0.97) and a significant increase in daily urine output [18]. Neither the PROTECTION trial nor the pooled meta-analysis demonstrated a significant reduction in 30-day mortality or the overall requirement for renal replacement therapy.

Subgroup analyses indicate that the protective effect is consistent across high-risk populations. In patients with pre-existing CKD (estimated glomerular filtration rate [eGFR] < 60 mL/min/1.73 m^2^), amino acid infusion achieved an absolute risk reduction of 7%, corresponding to a number needed to treat of 14 [61]. In patients requiring temporary mechanical circulatory support, the AKI rate was reduced by 30% with a number needed to treat of six [62]. Efficacy also correlates with the severity of the intraoperative renal insult: patients with CPB durations of 94 min or longer derived significantly greater benefit while those with shorter bypass exposure showed a non-significant effect, with progressive divergence in AKI probability between groups as CPB time increased [63].

The optimal duration of amino acid administration has not been established and is complicated by potentially discordant safety signals. Peak serum urea was significantly higher in the prolonged infusion group (78.2 vs. 54.0 mg/dL), and a post hoc analysis of the PROTECTION trial found discordant effects of infusion duration on intensive-care unit (ICU) mortality, as follows: a reduction from 1.9% to 1.0% with brief infusion (≤48 h) versus an increase from 2.5% to 3.5% with prolonged infusion (>48–72 h; interaction *p* = 0.04), despite a consistent renoprotective effect (interaction *p* = 0.89) [64]. This hypothesis-generating signal has not been independently replicated, and since infusion duration in the parent trial was determined by the clinical course (until RRT initiation, ICU discharge, death, or 72 h) rather than randomized, it is highly susceptible to confounding by indication; a causal effect cannot be inferred.

Because large studies have relied on serum creatinine and eGFR as the principal measures of renal protection, it remains uncertain whether the observed reductions in AKI incidence reflect true attenuation of tubular structural injury or predominantly a functional augmentation of the filtration rate that transiently masks creatinine-based diagnostic thresholds [60,65]. The absence of tubular damage biomarkers—specifically neutrophil gelatinase-associated lipocalin (NGAL), kidney injury molecule-1 (KIM-1), and the [tissue inhibitor of metalloproteinases-2] × [insulin-like growth factor binding protein 7] ([TIMP-2] × [IGFBP7]) product—from large trial reporting prevents any determination of whether amino acid therapy prevents structural nephron injury or acts solely through functional GFR augmentation [65]. The minimum effective dose required to recruit renal functional reserve without inducing clinically significant uremia has not been established prospectively [60]. Long-term follow-up data characterizing CKD progression beyond 90–180 days post-randomization are unavailable [60,61], and efficacy in patients with advanced CKD (eGFR < 30 mL/min/1.73 m^2^) remains unproven [18,60].

These results have been incorporated into the 2024 guidelines on cardiopulmonary bypass management [54]. A Class IIa recommendation with a Level of Evidence B has been assigned to the perioperative intravenous infusion of a balanced mixture of amino acids to reduce the incidence of CS-AKI. It is suggested that a 48-h infusion is sufficient to achieve renal protective effects. Given that the mortality signal linked to prolonged infusion is unreplicated and likely confounded by indication, limiting infusion to ≤48 h should be regarded as a prudent, guideline-consistent practice rather than a response to demonstrated harm.

Based on current data, perioperative amino acid infusion should be considered a standard renoprotective strategy in cardiac surgery, as it is supported by robust evidence demonstrating reductions in both overall and severe CS-AKI.

### 3.3. Anemia, Intraoperative Hemoglobin Tolerance and Transfusion Strategy

This section addresses two interventions of distinct evidentiary status that share a common physiological rationale—preoperative anemia correction and intraoperative liberal-versus-restrictive red cell transfusion strategy—which falls within the category of high-quality evidence of no renal benefit, with adequately powered multicenter randomized trials demonstrating equivalence rather than inconclusive results.

Hemoglobin concentration determines the oxygen-carrying capacity of blood and, in conjunction with cardiac output, defines DO_2_. During CPB, where flow is externally fixed, hemoglobin becomes the dominant modulator of DO_2_: reductions in hematocrit translate directly into reduced oxygen transport without the compensatory increases in heart rate or stroke volume available in the native circulation. The renal medulla, operating near its oxygen-extraction ceiling under physiological conditions, is the segment most vulnerable to this reduction. When DO_2_ falls below the critical threshold of approximately 260–280 mL/min/m^2^ during CPB, postoperative AKI increases measurably, and the anemic patient enters bypass already closer to that threshold than the nonanemic counterpart [8,9,66].

Preoperative anemia affects 16–54% of cardiac surgical patients depending on the definition and cohort characteristics [24]. In a meta-analysis of 114,277 patients, preoperative anemia tripled the risk of postoperative AKI (odds ratio [OR] 3.13; 95% CI 2.37–4.12) and was associated with a near-tripling of perioperative mortality (OR 2.74; 95% CI 2.32–3.24) [24]. At the single-center level, prospective data from 1047 consecutive on-pump coronary artery bypass grafting (CABG) patients confirmed preoperative anemia as an independent predictor of AKI by RIFLE criteria (OR 2.06; 95% CI 1.14–3.70) after adjustment for insulin-dependent diabetes and a propensity score for transfusion likelihood [66]. The propensity score for transfusion itself emerged as an independent AKI predictor in the same model (OR 2.23; 95% CI 1.80–2.80), indicating that renal risk from preoperative anemia operates at least partly through increased transfusion exposure rather than oxygen-delivery deficit alone.

Intraoperative relative hemoglobin decline carries independent prognostic weight that is not captured by absolute nadir values. In 11,508 cardiac surgical patients, a ≥50% reduction in hemoglobin during surgery was associated with increased risk of the composite of death, stroke, myocardial infarction, and renal failure (adjusted odds ratio [aOR] 1.27; 95% CI 1.14–1.41), even when nadir hemoglobin remained above 7 g/dL [67]. In a separate cohort of 10,179 patients with normal preoperative hemoglobin, a >50% intraoperative reduction was independently associated with death, stroke, or kidney failure, while nadir hemoglobin below 7–8 g/dL was not independently predictive after full risk adjustment [22]. The clinical implication is that a patient who hemodilutes from 14 g/dL to 8 g/dL—a 43% decline—may carry a lower risk than one who reaches the same nadir from a preoperative value of 12 g/dL through a 33% fall that crosses the relative threshold associated with impaired organ tolerance [22]. Data from a prospective cohort of 23,860 patients, including 270 Jehovah’s Witnesses who declined transfusion, found that outcomes in non-transfused Witnesses with nadir hemoglobin below 8 g/dL were comparable to those of propensity-matched non-transfused non-Witnesses, with only modest differences relative to patients receiving a single red blood cell unit [68].

The nephrotoxic potential of transfused red blood cells is independently documented. In 12,388 cardiac surgical patients, transfusion exposure demonstrated a dose-dependent association with AKI that was significantly stronger in preoperatively anemic patients—with AKI rising from 1.8% to 6.6% across zero to three transfused units in the anemic subgroup versus 1.7% to 3.2% in nonanemic patients—supporting the possibility of an interaction between baseline anemia and transfusion-associated renal injury [23]. In the TRIBE-AKI consortium, intraoperative transfusion was independently associated with dose-dependent elevations in urinary NGAL, interleukin-18 (IL-18), KIM-1, and liver fatty acid-binding protein (L-FABP), with patients receiving four or more units demonstrating 2.1-fold higher urinary NGAL concentrations than those receiving one to three units [69].

Randomized evidence does not support liberal transfusion as a renal-protective strategy. This finding was first established by the single-center TRACS trial, which demonstrated the noninferiority of a restrictive transfusion strategy (hematocrit [Hct] ≥ 24%) over a liberal strategy (Hct ≥ 30%) for a composite endpoint including AKI, with comparable renal outcomes between groups [70]. In TRICS III, 5243 high-risk cardiac surgical patients (EuroSCORE I ≥ 6) randomized to a restrictive threshold (Hb < 7.5 g/dL) versus a liberal threshold (Hb < 9.5 g/dL in the operating room or ICU, <8.5 g/dL on the ward) showed a similar incidence of the primary composite outcome—death, myocardial infarction, stroke, or new-onset dialysis (11.4% vs. 12.5%). Despite lower transfusion rates in the restrictive arm (52.3% vs. 72.6%), no significant differences were observed in individual secondary outcomes [25]. Pooled analysis of six RCTs confirmed no significant difference in CS-AKI incidence between strategies, with high-quality Grading of Recommendations Assessment, Development and Evaluation (GRADE) evidence supporting the absence of a meaningful effect in either direction [1].

Red blood cell administration during CPB is accordingly recommended only at a hematocrit below 18% (Hb < 6.0 g/dL; Class I, Level C) or between 18% and 24% when there is objective evidence of inadequate tissue oxygenation (DO_2_ below 273 mL/min/m^2^ or abnormal cerebral oximetry; Class IIb, Level B) [54].

Based on current data, preoperative anemia should be actively corrected whenever feasible, while a restrictive transfusion strategy is preferred, as liberal transfusion practices have not demonstrated renal benefit.

### 3.4. The KDIGO Bundle of Care

The kidney-protection bundle is a multicomponent preventive strategy that has demonstrated a significant reduction in moderate-to-severe CS-AKI, with a number needed to treat (NNT) of 12 in the definitive BigpAK-2 trial [20]. Its intraoperative relevance lies in risk stratification, as follows: urinary [TIMP-2]·[IGFBP7] (NephroCheck^®^), measured 2–4 h after cardiac surgery, identifies tubular cell-cycle arrest before serum creatinine or urine output reflect functional impairment, with an AUC of approximately 0.82–0.90 in cardiac surgical populations [19]. A value exceeding 0.3 (ng/mL)^2^/1000 identifies patients at elevated risk of stages 2–3 AKI and triggers protocolized bundle delivery [71].

The bundle comprises three postoperative pillars. Hemodynamic optimization targets are achieved with noradrenaline or dobutamine as required, and fluid responsiveness is reassessed every three hours [20,71]. Nephrotoxin avoidance includes discontinuation of angiotensin-converting-enzyme (ACE) inhibitors and angiotensin receptor blockers for at least 48 h postoperatively and avoidance of hydroxyethyl starch, gelatin, vancomycin, aminoglycosides, chloride-rich solutions, and radiological contrast for at least 72 h [20,71]. Glycemic control was targeted at 100–150 mg/dL in the trial protocols [71], though the 2026 KDIGO public review draft recommends a revised target of 140–180 mg/dL to minimize hypoglycemia risk in critically ill patients [53].

The bundle’s efficacy has been established across three sequential trials. The single-center PrevAKI trial first demonstrated that biomarker-guided implementation reduced overall AKI incidence from 71.7% to 55.1% and stages 2–3 AKI from 44.9% to 29.7% [19]. The multinational PrevAKI-Multicenter trial confirmed feasibility and reproduced the reduction in severe AKI—from 23.9% to 14.0% (absolute risk reduction [ARR] 10%; *p* = 0.034)—with 65.4% complete adherence in the intervention arm versus 4.2% under standard care [71]. BigpAK-2 (*n* = 1180; 34 European hospitals) established definitive efficacy: stages 2–3 AKI occurred in 14.4% versus 22.3% of controls (OR 0.57; 95% CI 0.40–0.79; NNT = 12), without an increase in adverse events [20]. An individual participant data meta-analysis of four RCTs confirmed a reduction in stages 2–3 AKI from 27.1% to 17.7% [21]. Intention-to-treat analyses have not demonstrated significant differences in renal replacement therapy requirement, ICU length of stay, or 90-day mortality [20,21]. The 2026 KDIGO Clinical Practice Guideline Public Review Draft for AKI and acute kidney disease (AKD) assigns a 1B recommendation to this multicomponent strategy in adult cardiac surgical patients at high biomarker-defined risk [53].

Routine adoption of biomarker-guided KDIGO bundles outside tertiary centers remains challenging. The strategy requires both a urinary [TIMP-2]·[IGFBP7] assay to enrich for high-risk patients and the infrastructure to deliver the bundle (functional hemodynamic monitoring, protocolized volume and glycemic control, nephrotoxin avoidance); although approved by the US Food and Drug Administration (FDA) and carrying Conformité Européenne (CE) marking, biomarker testing is limited by cost and availability, particularly in non-tertiary and low-resource settings.

Based on current data, biomarker-guided implementation of the KDIGO bundle should be adopted in patients at high risk for CS-AKI, supported by consistent findings across studies.

### 3.5. Remote Ischemic Preconditioning (RIPC)

Brief cycles of transient limb ischemia trigger systemic protective signals that converge on the mitochondrial permeability transition pore through reperfusion injury salvage kinase (RISK) and survivor-activating factor enhancement (SAFE) pathways, attenuating cell death during ischemia–reperfusion injury [72]. In human right atrial biopsies, RIPC prevented the 28% reduction in maximal mitochondrial respiration observed following aortic cross-clamping, providing direct subcellular evidence of protection in the cardiac surgical setting [73].

Across 31 randomized controlled trials, RIPC via transient limb ischemia is associated with a risk ratio for CS-AKI of 0.86 (95% CI 0.78–0.95) [1]. The central controversy remains the failure of the two largest multicenter trials—ERICCA and RIPHeart—to demonstrate clinical benefit in reducing AKI as an independent outcome or as a component of composite endpoints [26,27]. The divergence between pooled estimates and large-trial null results is itself informative: funnel plot asymmetry with a significant Egger test (*p* = 0.03) indicates a small-study effect, suggesting that the pooled RR of 0.86 is likely an overestimate of the true treatment effect [1].

A pharmacological interaction provides the most substantiated explanation for the discordance between small-trial and large-trial results. Subgroup analyses demonstrate that RIPC significantly reduces AKI incidence in patients maintained on volatile anesthetics (OR 0.57) but is rendered ineffective under propofol-based anesthesia (OR 0.96), with a statistically significant difference between anesthetic regimens (*p* = 0.028) [74]. This interaction was prospectively validated by Zarbock et al., who deliberately avoided propofol and maintained anesthesia with sevoflurane in 240 high-risk patients (Cleveland Clinic Score [CCS] ≥ 6); RIPC produced a significant reduction in AKI incidence (37.5% vs. 52.5%; *p* = 0.02) and in stages 2–3 AKI (12.5% vs. 25.8%; *p* = 0.02) [28]. Both ERICCA and RIPHeart employed propofol as the primary anesthetic agent, a drug reported to suppress β-adrenoreceptor responsiveness and interfere with signal transducer and activator of transcription 5 (STAT5) activation—pathways integral to the preconditioning stimulus [28,72]. The HypnoRenalRIP trial provided direct mechanistic confirmation: in patients maintained on sevoflurane, RIPC induced a significant increase in urinary [TIMP-2]·[IGFBP7] compared with sham–RIPC (*p* = 0.037), a response entirely absent under propofol (*p* = 0.755), with the absolute biomarker change significantly greater in the sevoflurane–RIPC group (median change 0.070 vs. –0.015; *p* = 0.022). The trial was designed and powered around this biomarker endpoint rather than clinical outcomes; the absence of significant differences in major adverse kidney events at 90 days accordingly reflects insufficient statistical power to detect effects on patient-centered outcomes rather than a definitive null result at the clinical level [75].

The optimal stimulus parameters have not been established by prospective dose-finding studies. Most trials apply RIPC immediately before surgery, potentially failing to exploit the second window of protection, which occurs 24–72 h after the initial stimulus and may confer more durable organ protection [1,72].

Guideline recommendations reflect these uncertainties. The Society of Thoracic Surgeons (STS), Society of Cardiovascular Anesthesiologists (SCA), and American Society of Extracorporeal Technology (AmSECT) acknowledge RIPC but do not issue a formal recommendation [76]. In contrast, the 2024 European Association for Cardio-Thoracic Surgery (EACTS), European Association of Cardiothoracic Anaesthesiology and Intensive Care (EACTAIC), and European Board of Cardiovascular Perfusion (EBCP) Guidelines on cardiopulmonary bypass in adult cardiac surgery state that RIPC may be considered in patients receiving volatile anesthesia to mitigate myocardial and kidney injury (Class IIa) [54].

Based on current evidence, RIPC may be considered to reduce myocardial and kidney injury in patients anesthetized with volatile agents rather than propofol. This anesthetic interaction is supported by post hoc subgroup analysis and, more recently, by the dedicated 2 × 2 factorial HypnoRenalRIP trial, in which the RIPC-associated biomarker response occurred only under sevoflurane. However, that trial was powered for a biomarker endpoint and showed no significant difference in clinical outcomes, so the interaction should be regarded as mechanistically supported rather than confirmed on hard clinical endpoints.

### 3.6. Pulsatile Flow During CPB

The transition from physiological pulsatility to continuous laminar flow during CPB reduces hemodynamic energy transmission to the vascular wall, impairing endothelial vasodilatory signaling and promoting microcirculatory shunting [77,78]. Despite this mechanistic rationale, consistent translation to clinically defined CS-AKI reduction has not been demonstrated. Earlier meta-analyses suggested a renal benefit with pulsatile perfusion [79,80], but a more rigorous analysis restricted to 10 RCTs (*n* = 1993) reported only a modest and heterogeneous reduction in CS-AKI incidence [1]. A large pragmatic study of 2489 patients found no association between universal pulsatile CPB and AKI incidence, suggesting that any benefit is attenuated in unselected populations by competing intraoperative determinants of renal injury [29].

The modality by which pulsatility is generated materially determines the clinical outcome. Post hoc subgroup analysis identified a significant AKI reduction with intra-aortic balloon pump (IABP)-generated pulsatility but not with pump-generated pulsatility, although the interaction test between subgroups did not reach statistical significance [1]. This pattern may reflect the superior waveform fidelity of IABP and the nephrotoxic counterbalance introduced by pump-generated pulsatility—pulsatile roller pumps generate significantly higher circuit pressures and plasma free heme concentrations after unclamping, offsetting any microcirculatory benefit [81,82]. IABP applicability remains restricted to patients meeting specific clinical indications. Current guidelines position pulsatile perfusion as a Class IIa, Level B recommendation for patients at elevated renal risk [54].

Based on current data, pulsatile perfusion flow during CPB should be considered to decrease the risk of AKI during cardiac surgery.

### 3.7. Minimally Invasive Extracorporeal Circulation

The proposed renal-protective effect of minimally invasive extracorporeal circulation (MiECC) rests on two mechanisms: reduced hemodilution from smaller priming volumes and attenuation of the systemic inflammatory response through reduced blood exposure to artificial circuit surfaces. In a propensity-matched cohort of 142 patients, the hemodilution rate was significantly lower in the MiECC group (16.8% vs. 18.8%) [83]. Whether these mechanistic advantages translate into a clinically measurable reduction in CS-AKI incidence remains unresolved according to the current evidence.

Reported renal effects vary substantially according to the study design and endpoint definition. A Bayesian network meta-analysis of 29 studies comparing MiECC, conventional extracorporeal circulation (CECC), and off-pump surgery reported a greater than 50% reduction in renal dysfunction odds with MiECC versus CECC, with MiECC associated with the lowest posterior median rates across all three comparators [30]. A subsequent systematic review of 42 RCTs found no significant reduction in renal failure as an isolated endpoint though a significant benefit emerged when renal failure was included within a composite endpoint with mortality, stroke, and myocardial infarction [84]. This pattern—composite benefit without independent renal effect—is a recurring limitation of the MiECC literature and complicates the attribution of any protective signal to renal mechanisms specifically.

Biomarker data provide mechanistic support but do not resolve the clinical question. Urinary NGAL, α-glutathione S-transferase, liver fatty acid-binding protein, and KIM-1 were significantly reduced following MiECC-assisted coronary revascularization, and Provaznik et al. reported, in a large retrospective propensity score-matched CABG cohort that included 5164 patients, lower postoperative renal failure and hemodialysis rates with MiECC compared with conventional CPB [31]. Peak creatinine was significantly reduced in the MiECC arm in a separate RCT of 150 patients [85].

The multicenter COMICS trial, terminated early due to the coronavirus disease 2019 (COVID-19) pandemic after the enrollment of 1071 patients, reported MiECC reduction in the 30-day composite of serious adverse events versus CECC, but no renal-specific benefit was demonstrated. Stage 3 AKI/hemofiltration was part of the primary composite endpoint; stage 3 AKI occurred numerically more often with MiECC (13 vs. 10 cases), and the trial was underpowered for individual component outcomes [32]. No between-group difference in renal insufficiency was identified in 114 diabetic patients in a separate retrospective series [86]. Absence of a standardized definition of renal failure across trials remains a major methodological limitation [84].

Current guidelines recommend MiECC over CECC on the grounds of improved biocompatibility and reduced transfusion requirement (Class IIa, Level B), with atrial fibrillation (AF) prevention and blood conservation rather than AKI reduction as the primary clinical justifications [54].

Based on current data, MiECC during CPB is recommended to decrease the risk of blood loss and hemodilution and should be considered to reduce AF during cardiac surgery not for reducing AKI rates.

### 3.8. Dexmedetomidine

Dexmedetomidine, a selective α2-adrenoceptor agonist, has been investigated as a pharmacological strategy to reduce CS-AKI incidence through attenuation of the perioperative stress response. In the most comprehensive meta-analysis to date (16 RCTs; *n* = 2882), perioperative dexmedetomidine reduced CS-AKI incidence by 42% (RR 0.58; 95% CI 0.37–0.91) [46], a finding consistent with two independent pooled analyses reporting reductions of 34–46% across comparable patient populations [87,88]. The most pronounced absolute benefit—a 29% reduction in AKI—was observed in patients undergoing surgery for infective endocarditis, a population characterized by high baseline inflammatory burden [44]. These findings remain inconsistent across large trials: the DECADE trial [43]—designed with postoperative delirium as its primary endpoint and AKI as a secondary outcome—reported neutral effects on AKI risk. More recently, the DOCS trial [45], a large multicenter placebo-controlled RCT of 1073 patients undergoing CPB-assisted cardiac surgery, also found no significant reduction in AKI defined by KDIGO criteria (36.8% vs. 40.8%; RR 0.85; 95% CI 0.66–1.08) or renal failure, defined as stage 3 AKI or AKI requiring RRT (2.05% vs. 2.61%; OR 0.79; 95% CI 0.35–1.75). Professional society guidelines have not endorsed dexmedetomidine as a renal-protective strategy pending confirmatory evidence [44]. The pattern of benefit—strongest in patients with a high inflammatory and catecholaminergic burden but inconsistent in broader cardiac surgical populations—suggests that dexmedetomidine is unlikely to function as a universal renoprotective agent. Rather, its efficacy may depend on whether sympathetic activation and inflammatory amplification are dominant drivers of injury within the individual CS-AKI phenotype.

Proposed mechanisms of renal protection include attenuation of the sympathoadrenal response, reduction in circulating catecholamines, and preservation of cortical perfusion through renal arteriolar vasodilation during CPB [89]. Available evidence suggests that the renoprotective effect may be dose- and duration-dependent. Subgroup analysis identified that doses exceeding 0.5 μg/kg were associated with significant AKI reduction, while moderate doses of 0.4 μg/kg/h produced inconsistent results [46]. Administration spanning both intraoperative and postoperative periods produced more consistent protection than intraoperative exposure alone [87]. The neutral DOCS trial used a moderate-dose regimen of 0.4 μg/kg/h for 12 h from induction, supporting the possibility that dose, duration, timing, and patient phenotype may modify the renal response.

The apparently contradictory profile of dexmedetomidine—sympatholytic renoprotection coupled with dose-dependent hemodynamic instability—reflects a narrow therapeutic window in which catecholaminergic and inflammatory renal injury are attenuated without crossing into flow-limiting hypotension. In cardiac surgical patients specifically, this risk appears more manageable than in general ICU populations: in an RCT of 108 patients undergoing aortic surgery, bradycardia and hypotension rates were comparable between dexmedetomidine and control groups [90]. Long-term renal outcomes—including progression to CKD or requirement for chronic dialysis—have not been reported in available trials [87,91].

Despite favorable meta-analytic signals, dexmedetomidine has not been incorporated into current guidelines for CS-AKI prevention, reflecting heterogeneity in dosing strategies, inconsistent AKI definitions across trials, neutral findings from large RCTs in which renal outcomes were secondary endpoints, and the absence of large pragmatic studies designed around standardized renal endpoints [54]. Larger confirmatory trials defining an optimal regimen and target phenotype are required before routine adoption can be recommended.

Based on current data dexmedetomidine may represent a promising adjunct for renal protection, particularly at higher doses, but heterogeneity among studies and neutral findings from large trials preclude a firm recommendation at present.

### 3.9. Intraoperative Mean Arterial Pressure (MAP) Targets

Intraoperative MAP augmentation belongs to the category of high-quality evidence of no renal benefit: adequately powered randomized trials have demonstrated the absence of effect rather than inconclusive or heterogeneous results. The rationale for augmenting MAP during cardiac surgery as a renoprotective strategy rests on the assumption that elevating perfusion pressure improves renal blood flow, particularly when arterial pressure falls below the lower limit of renal autoregulation [92]. During CPB, however, effective renal perfusion depends on pump flow, oxygen delivery, hemoglobin concentration, and microcirculatory integrity—not pressure alone.

Randomized trial data have consistently failed to support MAP augmentation as a renal-protective strategy during CPB. A meta-analysis of trials comparing MAP targets ≥ 65 mmHg with lower targets found no significant reduction in AKI incidence [35]. In high-risk patients, maintaining MAP at 75–85 mmHg with norepinephrine compared with 50–60 mmHg produced identical AKI rates [93]. Targeting MAP above 60 mmHg versus 47 mmHg failed to attenuate loss of glomerular filtration rate measured at four months postoperatively [92]. Excessive vasopressor-driven pressure augmentation may be actively harmful: an MAP target of 70–80 mmHg was associated with a significantly higher incidence of stage 2 AKI and creatinine doubling compared with low-target management, attributed to the detrimental effects of vasoconstrictor load on renal microcirculation [33]. This paradox is explained by the mechanistic distinction between flow-limited and pressure-limited renal perfusion: vasopressor-driven increases in MAP unaccompanied by maintained pump flow may raise systemic vascular resistance without improving—and potentially reducing—effective renal perfusion [35]. The absence of benefit from MAP augmentation during CPB does not support permissive hypotension in the broader perioperative period. Post-CPB hypotension was independently associated with de novo postoperative RRT, with aORs of 1.13 for each 10 min epoch of MAP < 55 mmHg and 1.12 for MAP 55–64 mmHg; no association was observed before or during CPB [34]. In addition, MAP < 65 mmHg outside CPB has been associated with a composite adverse outcome that included AKI [94]. These data indicate that renal vulnerability to hypotension is concentrated in phases of restored pulsatile flow, where autoregulatory mechanisms re-engage and pressure–flow relationships resume physiological significance.

The principal evidence gap is the absence of trials individualizing MAP targets to patient-specific autoregulatory limits. Renal autoregulation operates across a pressure range of approximately 40 to 160 mmHg with substantial interindividual variability [92,94], yet available trials impose uniform absolute targets that may be subthreshold in some patients and suprathreshold in others. High-certainty data evaluating whether targeting MAP above individualized autoregulation-derived optimal pressures reduces CS-AKI incidence are not available [54]. Current guidelines recommend maintaining an intraoperative MAP between 50 and 80 mmHg while prioritizing goal-directed perfusion and oxygen delivery targets over pharmacological pressure augmentation for renal protection (Class I, Level A) [54].

Based on current data, a target of MAP > 80 mmHg with vasopressors during CPB is not recommended to decrease the risk of AKI.

### 3.10. Volatile Anesthetic Agents vs. Total Intravenous Anesthesia

Anesthetic agent selection—volatile vs. total intravenous anesthesia (TIVA)—likewise falls within the category of high-quality evidence of no renal benefit, where pragmatic multicenter trials have ruled out a clinically meaningful difference rather than leaving the question open. Volatile halogenated ethers exert cardioprotective effects through activation of the RISK (Reperfusion Injury Salvage Kinase) and SAFE (Survivor Activating Factor Enhancement) pathways converging on mitochondrial permeability transition pores to enhance cellular survival during ischemia–reperfusion injury [95]. This mechanistic basis has not translated into demonstrable renal protection in clinical cardiac surgery. Unlike the myocardium, the kidney responds adversely to volatile anesthesia: experimental data demonstrate greater reductions in renal blood flow and medullary perfusion with volatile agents than with propofol, accompanied by activation of renal sympathetic nerve activity and the renin-angiotensin-aldosterone system—a neurohormonal vasoconstrictor state not observed under propofol anesthesia [37,96].

In cardiac surgery, current evidence does not support a definitive renal-protective recommendation for either volatile-based anesthesia or propofol-based total intravenous anesthesia. The large MYRIAD trial [36] in elective isolated CABG found no significant difference in major clinical outcomes between volatile anesthesia and total intravenous anesthesia, while AKI and RRT were assessed only as prespecified adverse events rather than primary renal endpoints. Conversely, a mixed-surgical meta-analysis of 15,140 patients suggested lower postoperative AKI with propofol (random-effects OR 0.49; 95% CI 0.33–0.73), but most included patients underwent non-cardiac surgery, limiting direct applicability to cardiac surgery. More recently, a multicenter RCT of 3083 cardiac surgery patients found no benefit of volatile anesthesia over propofol-based TIVA for the composite primary outcome of major complications and 30-day mortality, which included renal failure, with similar event rates between groups [97].

The pharmacological confound of concurrent propofol administration likely explains a substantial part of this heterogeneity. Propofol inhibits the intracellular protective pathways through which volatile agents are presumed to act [95], and its use was prevalent in both the induction (89.1%) and maintenance phases (58.8%) of the volatile arms in the pragmatic trials reporting null results [36]. A pilot study randomizing higher-risk patients (EuroSCORE ≥ 5) to a propofol-free total inhalational strategy demonstrated feasibility, though it was not powered to detect differences in clinical outcomes [95]. Current guidelines assign volatile agents for maintenance during CPB a Class IIb, Level B recommendation [54], and the STS/SCA/AmSECT guidelines do not include anesthetic agent selection among their recommendations for CS-AKI prevention [76].

Based on current data, the use of intraoperative volatile anesthetics may be considered for the maintenance of anesthesia during CPB.

### 3.11. N-Acetylcysteine

The generation of reactive oxygen species during ischemia–reperfusion injury, acting in concert with neurohormonal activation and hemodilution-induced hypoxia, provides the mechanistic rationale for antioxidant intervention in CS-AKI [98]. N-acetylcysteine (NAC), a glutathione precursor with inhibitory effects on reactive oxygen species (ROS)-mediated and apoptotic signaling pathways, has been the most extensively evaluated agent in this context. Its renal benefit appears restricted to patients with pre-existing renal insufficiency, in whom pooled analysis demonstrates a significant reduction in CS-AKI incidence with intravenous administration (RR 0.77; 95% CI 0.63–0.94), while across unselected adult cardiac surgery populations no significant overall reduction has been demonstrated [39,98].

Administration route and dose appear to be important modifiers of NAC efficacy. In Zhao et al.’s meta-analysis [39], oral NAC showed no significant benefit on renal outcomes, possibly owing to first-pass metabolism and lower plasma concentrations, whereas intravenous NAC was associated with a modest reduction in AKI incidence (RR 0.84; 95% CI 0.71–0.99). However, the overall effect on AKI was neutral and trial sequential analysis (TSA) remained inconclusive, with no reduction in RRT. Within intravenous regimens, only high-dose administration—a 150 mg/kg loading dose followed by continuous infusion—achieves significant renoprotection while standard-dose protocols do not [38]. Neither regimen has reduced all-cause mortality or renal replacement therapy requirement in pooled analyses.

The inconsistency across the NAC literature is unlikely to reflect simple trial heterogeneity. Renal protection by NAC probably depends on the simultaneous interaction of at least four patient-level variables, as follows: timing of administration relative to ischemic onset, dose adequacy, baseline oxidative burden, and pre-existing renal function. Patients with CKD carry a higher oxidative load at baseline and a narrower physiological reserve, which may explain why this subgroup responds while general surgical populations do not. The dose–response pattern—oral failure, standard intravenous (IV) neutrality, and high-dose IV efficacy—is consistent with a threshold effect rather than a graded one, suggesting that most published trials operated below the pharmacologically active range. That the benefit signal is most reproducible precisely where oxidative stress is highest and dosing most aggressive supports this interpretation, though it remains inferential in the absence of a prospective dose-finding trial [38,39,98].

The European guidelines assign perioperative intravenous NAC a Class IIb, Level B recommendation for reducing AKI incidence in patients with pre-existing chronic kidney disease [54]. The American guidelines do not include NAC among their recommendations for CS-AKI prevention—not even with a weak recommendation—reflecting the absence of sufficiently robust evidence to support its routine adoption in unselected cardiac surgical populations [76].

Based on current data, intravenous NAC may be considered to decrease the risk of AKI.

### 3.12. Levosimendan

Levosimendan enhances renal hemodynamics through adenosine-triphosphate (ATP)-sensitive potassium-channel-mediated vasodilation of preglomerular resistance vessels. In patients with preserved renal function undergoing cardiac surgery, a loading dose of 12 μg/kg followed by 0.1 μg/kg/min increased GFR by 21% and renal blood flow by 12% without compromising renal oxygenation [99]. When administered to patients with established stages 1–2 AKI; however, renal blood flow remained responsive (15% increase) while GFR failed to improve significantly, suggesting that established tubular injury may negate the hemodynamic benefit [100].

Early meta-analyses generated a consistent renal signal: a pooled analysis of 13 RCTs reported a 49% reduction in AKI incidence (OR 0.51; 95% CI 0.34–0.76) and a 57% reduction in renal replacement therapy requirement (OR 0.43; 95% CI 0.25–0.76; *p* = 0.002) [47].

Elbadawi et al. reported a 41% relative reduction in study-defined postoperative AKI with prophylactic levosimendan (RR 0.59; 95% CI 0.38–0.92), but AKI definitions were not standardized and postoperative dialysis was not significantly reduced (RR 0.71; 95% CI 0.43–1.16) [101]. In LEVO-CTS, prophylactic levosimendan did not significantly reduce 30-day RRT requirement (2.1% vs. 3.8%; OR 0.54; 95% CI 0.24–1.24) [48]. The LICORN trial similarly found no renal benefit in patients with left ventricular ejection fraction (LVEF) ≤ 40% undergoing CABG: RRT was not reduced and was numerically higher in the levosimendan arm (9% vs. 6%; *p* = 0.16), with no mortality benefit at 180 days [102]. Similarly, a Cochrane review of nine studies reported little or no difference in renal outcomes, with low-certainty evidence [49].

The European guidelines assign levosimendan a Class IIa, Level A recommendation for perioperative use in patients with reduced LVEF undergoing isolated CABG, and a Class IIb, Level B recommendation for established low cardiac output syndrome (LCOS)—both indications based on hemodynamic rather than renal endpoints [54]. Levosimendan is not included among the guideline’s renal protection recommendations, and neither the STS/SCA/AmSECT nor the KDIGO framework endorse its use for CS-AKI prevention [76].

Based on current data, levosimendan should be considered for isolated CABG patients with LVEF < 40% to reduce AKI, RRT and LCOS.

### 3.13. Extracorporeal Blood Purification with the oXiris Membrane

The oXiris membrane is distinguished among hemoadsorption devices by its dual capacity to adsorb circulating cytokines and bind endotoxins, providing a mechanistic rationale for its use during CPB where complement activation, neutrophil priming, and cytokine release contribute to tubular injury [40]. The principal clinical evidence derives from the SIRAKI02 trial, in which intraoperative oXiris use reduced CS-AKI incidence from 39.7% to 28.4%, though this reduction was attributable to early stage 1 events defined by urine output criteria rather than creatinine, and was not accompanied by differences in vasopressor requirement, ventilation duration, or ICU length of stay [40]. Sensitivity analyses suggested greater benefit in patients with diabetes, hypertension, and reduced left ventricular ejection fraction below 40%, consistent with the hypothesis that cytokine adsorption is most effective where baseline inflammatory burden is highest, though these subgroup findings are post hoc [40].

Samaniego-Laguna et al. [42] reported that intraoperative hemoadsorption during CPB reduced reported AKI incidence in RCTs, but without significant effects on RRT, mortality, ICU/hospital stay, or mechanical ventilation. The finding should be interpreted cautiously because AKI definitions were heterogeneous, and the AKI signal was influenced by the REMOVE trial [103]. A separate synthesis of 17 studies—7 RCTs and 10 observational cohorts (*n* = 1797)—found that the apparent renal benefit was confined to observational data, with pooled RCT evidence showing no significant AKI reduction [41]. Once the REMOVE trial was excluded, the remaining studies became fully consistent with one another, suggesting that part of the statistical discordance may reflect the biological distinctiveness of the endocarditis population rather than a genuine heterogeneity of the treatment effect. The consistent finding that the benefit is driven by non-randomized data—where hemoadsorption was preferentially applied to higher-risk patients—substantially limits causal inference.

According to European guidelines, hemoadsorption is not recommended for elective cardiac surgery (Class III, Level B), while its use may be considered in infective endocarditis, where endotoxemia provides a more specific substrate for the dual mechanism of the oXiris membrane (Class IIb, Level B) [54].

Based on current data, oXiris membrane may be considered to decrease the risk of AKI during CPB, especially in patients with endocarditis.

### 3.14. Natriuretic Peptides: ANP

Atrial natriuretic peptide (ANP) has been investigated as a pharmacological strategy for perioperative renal protection because of its natriuretic, vasodilatory, and renin–angiotensin–aldosterone system-suppressing effects. During cardiopulmonary bypass, these actions may attenuate neurohormonal activation, preserve renal perfusion, and reduce creatinine-based renal dysfunction. Among drug-based strategies evaluated for CS-AKI prevention, ANP and related natriuretic peptides have generated one of the strongest pooled renal signals, although the evidence remains dominated by relatively small trials, heterogeneous AKI definitions, and Japanese carperitide experience [51].

The main trial-level evidence derives from the NU-HIT trial, where 504 CABG patients without preoperative renal impairment were randomized to low-dose human atrial natriuretic peptide (hANP)/carperitide infusion from the initiation of CPB or placebo. hANP preserved creatinine clearance and reduced several markers of postoperative renal dysfunction, including serum creatinine (sCr) increase ≥ 0.3 mg/dL (40 vs. 92 patients; *p* < 0.0001) and Cr > 2.0 mg/dL (1 vs. 8 patients; *p* = 0.0374). Hemodialysis occurred less frequently with hANP but was not statistically significant [104]. The subsequent NU-HIT CKD trial enrolled non-dialysis CKD patients undergoing on-pump CABG. Carperitide reduced early postoperative dialysis (1/141 vs. 8/144; *p* = 0.0362), total dialysis up to 1 year (2 vs. 13 patients; *p* = 0.0060), and improved 1-year dialysis-free rate (98.6% vs. 91.6%; *p* = 0.0066). Creatinine-based renal injury was also reduced, while sCr and eGFR remained more favorable throughout 1-year follow-up [50].

Pooled analyses have reproduced this renal signal but also define its limitations. In the systematic review and meta-analysis by Yamada et al., 18 RCTs of low-dose ANP ≤ 50 ng/kg/min were included, comprising 16 prevention and 2 treatment trials across mixed AKI-risk settings. In prevention RCTs, low-dose ANP reduced new AKI (RR 0.51; 95% CI 0.36–0.72; *p* = 0.0001) and RRT requirement (RR 0.17; 95% CI 0.04–0.64; *p* = 0.009). However, AKI definitions varied across trials, most studies had high or unclear risk of bias, and trial sequential analysis indicated insufficient cumulative information size for most outcomes. The AKI effect was also attenuated after exclusion of the two large Sezai-group trials indicating substantial dependence on single-center Japanese data [105].

In a Bayesian network meta-analysis of 95 RCTs including 28,833 patients and 13 pharmacological interventions, atrial natriuretic peptide was associated with lower postoperative renal dysfunction compared with placebo (OR 0.28; 95% credible interval (CrI) 0.17–0.48; moderate-quality evidence) and lower hemodialysis requirement (OR 0.24; 95% CrI 0.10–0.58; low-quality evidence). ANP was ranked highest for renal dysfunction and hemodialysis, and the effect remained apparent in the subgroup with normal baseline renal function. These estimates were limited by non-uniform renal definitions, indirect comparisons, and the assumptions inherent to network meta-analysis, since renal dysfunction was defined by study-specific criteria across the contributing trials [51]. In the broader perioperative meta-analysis by Pathak et al., atrial natriuretic peptides were evaluated in 14 trials including 2207 patients and included nesiritide, carperitide, and synthetic ANP. ANP reduced study-defined AKI and was associated with reduced 30-day or hospital mortality in the primary analysis. In sensitivity analyses restricted to trials at low risk of allocation-concealment bias, the AKI and RRT signals persisted, whereas the mortality effect did not. Certainty was rated low for mortality, moderate for AKI, and high for RRT [52].

The discrepancy between favorable pooled renal estimates and lack of routine clinical adoption reflects limitations that aggregate analyses cannot remove. Most ANP trials were performed in Japan, intravenous ANP formulations are not licensed or commercially available in Europe, and external validity to contemporary Western cardiac surgical populations remains uncertain [52]. The 2012 KDIGO guideline recommended against ANP for AKI prevention or treatment despite promising pilot data, and the intervention has not been incorporated into STS/SCA/AmSECT recommendations for CS-AKI prevention [55]. ANP, therefore, remains a biologically plausible and meta-analytically favorable intervention constrained by geographical concentration of evidence, formulation availability, heterogeneous renal endpoints, and absence of a large pragmatic multicenter trial using contemporary KDIGO-defined CS-AKI outcomes.

Based on current data, use of natriuretic peptide should be considered to reduce the risk of AKI (moderate) and RRT (high certainty).

## 4. Conclusions

Cardiac surgery-associated acute kidney injury remains a frequent, multifactorial complication whose impact extends well beyond transient creatinine elevation to encompass renal replacement therapy, progression to CKD, long-term mortality, and substantial healthcare cost. Because it reflects the interaction of multiple perioperative insults rather than a single cause, no isolated intervention confers universal protection, and prevention is best framed as a multimodal, patient-centered process. This integrates preoperative risk stratification and optimization of anemia, volume status, and nephrotoxin exposure; intraoperative preservation of renal oxygen delivery during cardiopulmonary bypass through goal-directed perfusion, patient blood management, and—most promisingly—amino acid infusion; and postoperative biomarker-guided KDIGO care bundles. In contrast, remote ischemic preconditioning, pulsatile flow, minimally invasive extracorporeal circulation, dexmedetomidine, N-acetylcysteine, levosimendan, hemoadsorption, and natriuretic peptides yield only variable, subgroup-dependent signals, whereas liberal transfusion, routine MAP augmentation, and volatile anesthesia show no renoprotective benefit. Future large pragmatic trials with standardized definitions and clinically meaningful renal endpoints are needed; until then, structured implementation of evidence-based perioperative bundles remains the most rational approach.

## Figures and Tables

**Table 1:** Landmark Studies Reporting AKI as an Outcome in CS-AKI Prevention.

Trial/Author (Year)	*n*	Design	Country	Intervention vs. Comparator	AKI Endpoint	Key CS-AKI Result
Goal-Directed Perfusion						
de Somer 2011 [14]	359	Cohort	Belgium and UK	Nadir DO_2_i < 262 vs. ≥262 mL/min/m^2^	AKI stage 2 by AKIN sCr criteria	RISK SIGNAL—Critical DO_2_ threshold identified: nadir DO_2_i < 262, independent risk factor for AKI stage 2 (23.2% vs. 7.4%; OR 3.11, CI 95% 1.53–6.32)
GIFT—Ranucci 2018 [15]	326	RCT	International	DO_2_i ≥ 280 mL/min/m^2^ vs. usual care	AKIN creatinine criteria	POSITIVE—AKI reduction: AKIN stage 1 (11.5% vs. 22.4%; RR 0.45, CI 95% 0.25–0.83) and any AKI (15.4% vs. 24.7%; RR 0.55, CI 95% 0.32–0.97) but not AKIN stages 2–3 AKI
Mukaida 2023 [16]	300	RCT	Japan	DO_2_i ≥ 300 mL/min/m^2^ vs. conventional fixed-flow perfusion	KDIGO creatinine criteria	POSITIVE—Overall, AKI was reduced: 14.6% vs. 30.4% RR 0.48 (CI 95% 0.30–0.77), mainly through lower stage 1 AKI; no significant reduction in AKI stages 2–3
Amino Acid Infusion						
PROTECTION—Landoni 2024 [17]	3511	RCT	International	AA vs. placebo	KDIGO creatinine criteria; RRT	POSITIVE—AKI reduction: 26.9% vs. 31.7%, RR 0.85 (CI 95% 0.77–0.94); stage 3 AKI halved RR 0.56 (CI 95% 0.35–0.87). RRT was numerically lower but not significant
Jiang MA 2025 [18]	5059	SR/MA	International	AA vs. placebo	KDIGO, RIFLE, Brussels Scale	POSITIVE—AKI reduction: RR 0.81 (CI 95% 0.68–0.97; I^2^ = 41%) but did not reduce RRT
Biomarker-Guided KDIGO Care Bundle						
PrevAKI—Meersch 2017 [19]	276	RCT	Germany	KDIGO bundle vs. standard care ([TIMP-2]·[IGFBP7] ≥ 0.3)	KDIGO overall AKI; stages 2–3 AKI	POSITIVE—Severe AKI reduction: overall 55.1% vs. 71.7% OR 0.483 (CI 95% 0.293–0.796); stages 2–3: 29.7% vs. 44.9% OR 0.518 (CI 95% 0.316–0.851)
BigpAK-2—Zarbock 2025 [20]	1180	RCT	Europe	KDIGO bundle vs. standard care	KDIGO stages 2–3 AKI within 72 h	POSITIVE—Severe AKI reduction: 14.4% vs. 22.3% (OR 0.57, CI 95% 0.40–0.79)
von Groote 2026 [21]	1851	MA (4 RCTs)	International	KDIGO bundle vs. standard care	KDIGO stages 2–3 AKI	POSITIVE—Severe AKI reduction: 17.7% vs. 27.1% (OR 0.55; CI 95% 0.44–0.70)
Preoperative Anemia Management						
Karkouti 2008 [22]	10,179	Cohort	Canada	>50% intraoperative Hb reduction vs. lesser decline	SCr x ≥ 2	RISK FACTOR—Relative Hb decline, not nadir value alone, determines AKI risk: aOR 1.53 (CI 95% 1.12–2.08)
Karkouti 2011 [23]	12,388	Cohort	Canada	Transfusion in anemic vs. non-anemic patients	RIFLE	RISK FACTOR—Synergistic AKI risk in anemic patients receiving transfusion: AKI 1.8%→6.6% vs. 1.7%→3.2% (p-interaction = 0.0007)
Padmanabhan 2019 [24]	114,277	MA (22 studies)	International	Preoperative anemia vs. no anemia	AKI	RISK FACTOR—Preoperative anemia triples AKI risk: OR 3.13 (CI 95% 2.37–4.12)
Transfusion Strategy (Restrictive vs. Liberal)						
TRICS III—Mazer 2017 [25]	5243	RCT	International	Restrictive (Hb < 7.5 g/dL) vs. liberal (Hb < 9.5 g/dL)	Composite endpoint: including RRT	NEUTRAL—No AKI benefit from liberal transfusion strategy; noninferiority confirmed: 11.4% vs. 12.5% OR 0.90 (CI 95% 0.76–1.07)
Hariri 2023 [1]	8289	MA (6 RCTs)	International	Restrictive vs. liberal transfusion	Consensus: AKI, KDIGO, RIFLE	NEUTRAL—Evidence of no effect in either direction: RR 1.02 (CI 95% 0.92–1.12)
Remote Ischemic Preconditioning						
ERICCA—Hausenloy 2015 [26]	1612	RCT	UK	RIPC vs. Sham (mixed propofol/volatile)	KDIGO	NEGATIVE—No AKI reduction: 38.0% vs. 38.3% (*p* = 0.98)
RIPHeart—Meybohm 2015 [27]	1403	RCT	Germany	RIPC vs. Sham (propofol)	ARF: sCr ≥ 2× or UO ≤ 0.5 mL/kg/h for 12 h, or RRT, or renal failure on autopsy	NEGATIVE—No AKI reduction: 6.1% vs. 5.1%, OR 0.83 (CI 95% 0.52–1.34)
Zarbock 2015 [28]	240	RCT	Germany	RIPC vs. sham (sevoflurane)	KDIGO	POSITIVE—AKI reduction: 37.5% vs. 52.5% (absolute risk reduction 15%; RR 0.71; CI 95% 0.54–0.95). KDIGO stages 2–3 reduction: 12.5% vs. 25.8% (*p* = 0.02)
Hariri 2023 [1]	7738	MA (31 RCTs)	International	RIPC vs. sham	Consensus: AKI, KDIGO, RIFLE	POSITIVE MODEST SIGNAL—Pooled AKI reduction likely overestimated due to small-study bias: 22% vs. 24.2%, RR 0.86 (CI 95% 0.78–0.95); Egger *p* = 0.03
Pulsatile Flow During CPB						
Coulson 2020 [29]	2489	Cohort	UK	Universal pulsatile CPB vs. non-pulsatile	KDIGO	NEGATIVE—No AKI benefit from universal pulsatile CPB in unselected population: 23.9% vs. 25.4% OR 1.09 (CI 95% 0.89–1.33). No differences in AKI staging or subgroup analyses (prolonged CPB, CKD)
Hariri 2023 [1]	1993	MA (10 RCTs)	International	Pulsatile vs. non-pulsatile CPB	Consensus: AKI, KDIGO, RIFLE	POSITIVE—Significant pooled AKI reduction: RR 0.69 (CI 95% 0.48–0.99); high heterogeneity and methodological concerns
Minimally Invasive Extracorporeal Circulation						
Kowalewski 2016 [30]	13,791	Network MA (134 RCTs)	International	MiECC vs. CECC vs. off-pump	Renal dysfunction (variable definitions across RCTs)	POSITIVE—MiECC associated with lowest renal dysfunction rates vs. CECC: OR 0.47 (CI 95% 0.24–0.89). SUCRA ranking: MiECC > OPCAB > CECC
Provaznik 2020 [31]	5164	Propensity score-matched analysis	Germany	MiECC vs. CECC	RRT	POSITIVE— CECC was independently associated with a higher risk of postoperative dialysis compared with minimized MiECC: 5.9% vs. 3.5% OR 1.74 (CI 95% 1.10–2.76)
COMICS—Angelini 2025 [32]	1071	RCT	International	MiECC vs. CECC (terminated early due to COVID)	SAES/stage 3 AKI/RRT	POSITIVE (composite)/NEUTRAL (AKI individually)—MiECC reduced composite SAEs by ~25%: RR 0.732 (CI 95% 0.556–0.962); however, individual AKI events were few (13 vs. 10)
Intraoperative MAP Targets						
Vedel 2018 [33]	197	RCT	Denmark	MAP 70–80 vs. 40–50 mmHg	Stage 2 AKI; creatinine doubling	NEGATIVE—Higher MAP target paradoxically increases severe AKI risk: stage 2 AKI RR 4.64 (CI 95% 1.03–20.93); creatinine doubling 9% vs. 2% (CI 95% 1.03–23.32)
Ngu 2020 [34]	6523	Cohort	Canada	MAP < 55 and 55–64 post-CPB (each 10 min epoch)	RRT	RISK SIGNAL—Post-CPB hypotension independently associated with RRT: MAP < 55 mmHg aOR 1.13 (CI 95% 1.05–1.23) and MAP 55–64 mmHg aOR 1.12 (CI 95% 1.06–1.18) per 10 min epoch
Kotani 2022 [35]	487	MA (2 RCTs)	International	MAP ≥ 65 mmHg vs. <65 mmHg during CPB	AKI RIFLE/KDIGO	NEUTRAL—No AKI reduction with higher MAP targets across pooled RCTs: RR 1.30 (CI 95% 0.81–2.08)
Volatile Anesthesia vs. TIVA						
MYRIAD—Landoni 2019 [36]	5400	RCT	International	Volatile vs. TIVA	RIFLE	NEGATIVE—No renal benefit from volatile anesthesia in largest multicenter RCT: AKI 1.4% vs. 1.3% (RR 1.11; ns)
Franzén 2023 [37]	15,140	MA (8 studies)	International	Propofol vs. volatile	KDIGO, AKIN, RIFLE	POSITIVE (propofol)—Propofol associated with significantly lower AKI incidence: OR 0.49 (CI 95% 0.33–0.73)
N-Acetylcysteine						
Santana-Santos 2014 [38]	70	RCT	Brazil	High-dose IV NAC vs. placebo	AKIN criteria within 72 h	POSITIVE—AKI reduction in patients with pre-existing CKD: 28.6% vs. 57.1% (*p* = 0.016)
Zhao 2022 [39]	2444	MA 25 (RCTs)	International	NAC vs. control; IV vs. oral subgroup	Different definitions across RCTs, mostly by postoperative sCr increase > 25%; RRT also assessed.	NEUTRAL (overall)/IV signal—No overall AKI reduction (RR 0.91; CI 95% 0.77–1.08); TSA inconclusive. IV NAC reduced AKI modestly (RR 0.84; CI 95% 0.71–0.99), but oral NAC did not. No RRT benefit
Extracorporeal Blood Purification						
SIRAKI02—Pérez-Fernández 2024 [40]	343	RCT	Spain	oXiris during CPB vs. standard care	KDIGO	POSITIVE—AKI reduction: 28.4% vs. 39.7% (*p* = 0.03); benefit concentrated in mild AKI stages; no significant differences in RRT. Subgroup analyses suggested greater benefit in patients with CKD, diabetes, hypertension, low LVEF, and BMI < 30
Salles 2026 [41]	1797	MA (7 RCTs + 10 obs.)	International	Hemoadsorption during CPB vs. standard care	KDIGO, AKI, RRT	NEGATIVE (RCTs)—RCT evidence does not support AKI reduction: apparent benefit confined to observational data with selection bias
Samaniego-Laguna 2026 [42]	1133	MA (16 RCTs)	International	Hemoadsorption during CPB vs. standard care	KDIGO	WEAK SIGNAL—Significant pooled AKI reduction: RR 0.75 (CI 95% 0.59–0.96); however, no benefit in RRT, mortality, or any secondary endpoint; clinical relevance uncertain given dissociation between AKI signal and hard outcomes
Dexmedetomidine						
DECADE—Turan 2020 [43]	798	RCT	USA	Dexmedetomidine escalating dose vs. placebo	AKIN	NEGATIVE—No AKI benefit: significant hypotension (57% vs. 36%) may have offset any renoprotective effect
Ham 2024 [44]	63	RCT (interim analysis, stopped early)	South Korea	Dexmedetomidine 0.4 μg/kg/h × 24 h vs. placebo	KDIGO	POSITIVE—AKI reduction in IE population: 9.4% vs. 32.3%, RD −22.9 (CI 95% −42.2 to −3.6)
DOCS—Lei 2026 [45]	1073	RCT	China	Dexmedetomidine 0.4 μg/kg/h × 12 h vs. placebo	AKI by KDIGO; renal failure = stage 3 AKI or RRT (all as secondary outcomes)	NEUTRAL—No AKI reduction: 36.8% vs. 40.8%, RR 0.85 (CI 95% 0.66–1.08); renal failure: 2.05% vs. 2.61%, OR 0.79 (CI 95% 0.35–1.75)
Wen 2026 [46]	2882	MA (16 RCTs)	International	Dexmedetomidine vs. placebo	RIFLE, KDIGO, others	POSITIVE—Overall AKI reduction: RR 0.58 (CI 95% 0.37–0.91; I^2^ = 74%); benefit driven by high-dose subgroup (0.6–1.0 μg/kg/h): RR 0.43 (CI 95% 0.26–0.71; I^2^ = 0%); 0.4 μg/kg/h, NOT significant: RR 0.65 (CI 95% 0.36–1.17)
Levosimendan						
Zhou 2016 [47]	1345	MA (13 RCTs)	International	Levosimendan vs. placebo/or active inotropic control	sCr: relative increase 50%/absolute increase 0.3 mg/dL/level 1.5 mg/dL, or relative decrease in estimated GFR of 25% within 7 days	POSITIVE—AKI reduction: 15.6% to 8.7%, OR 0.51 (CI 95% 0.34–0.76) and RRT 10% to 4.5%, OR 0.43 (CI 95% 0.25–0.76) but evidence was limited by small trials, heterogeneous AKI definitions, unclear bias risk, and active comparators
LEVO-CTS—Mehta 2017 [48]	849	RCT	USA/Canada	Levosimendan vs. placebo	Composite, including death, RRT, MI, or mechanical cardiac assist device; RRT through 30 days	NEGATIVE—No reduction in the primary composite endpoint; RRT 30 days not significant: 2.1% vs. 3.8%, OR 0.54 (CI 95% 0.24–1.24)
Cochrane—Gayatri 2024 [49]	1819	Cochrane SR/MA, 9 studies for renal failure outcome	International	Levosimendan vs. placebo	sCr ≥ 0.3 mg/dL, diuresis < 0.5 mL/kg/h × 6 h; RRT	NEUTRAL—No clear reduction in renal failure: 4.7% vs. 6.7%; RR 0.71 (CI 95% 0.43–1.16; I^2^ = 30%.
Natriuretic Peptides (ANP)						
NU-HIT CKD—Sezai 2011 [50]	303	RCT	Japan	Carperitide vs. placebo (CKD stages 1–3)	Dialysis-free rate; sCr/eGFR; sCr increase ≥ 0.3 mg/dL	POSITIVE—hANP reduced dialysis up to 1 year (2 vs. 13; *p* = 0.0060), improved dialysis-free survival (98.6% vs. 91.6%; *p* = 0.0066), lowered maximum sCr, and reduced sCr increase ≥ 0.3 mg/dL (26% vs. 59%; *p* < 0.0001), with renal protection sustained at 1 year
Kim 2018 [51]	28,833 overall; ANP: 1213	Network MA (95 RCTs)	International	ANP vs. placebo	RIFLE, AKIN, KDIGO, others, dialysis	POSITIVE—ANP reduced postoperative renal dysfunction, OR 0.28 (CrI 95% 0.17–0.48; moderate-quality evidence) and hemodialysis OR 0.24 (CrI 95% 0.10–0.58; low-quality evidence)
Pathak 2021 [52]	2207	SR/MA; ANP subgroup from 14 RCTs	International	hANP vs. control	Study-defined AKI; RRT	POSITIVE—hANP reduced AKI (RR 0.43, CI 95% 0.33–0.56; I^2^ = 0%) and RRT (RR 0.26, CI 95% 0.15–0.47; I^2^ = 0%)

AKI = acute kidney injury; ARF = acute renal failure; CECC = conventional extracorporeal circulation; CKD = chronic kidney disease; CPB = cardiopulmonary bypass; CS-AKI = cardiac surgery-associated AKI; DO2 = oxygen delivery index; GFR = glomerular filtration rate; Hb = hemoglobin; Hct = hematocrit; hANP = human atrial natriuretic peptide; Int. MA = international meta-analysis; KDIGO = Kidney Disease Improving Global Outcomes; LVEF = left ventricular ejection fraction; MA = meta-analysis; MiECC = minimally invasive extracorporeal circulation; MI = myocardial infarction; ns = non-significant; OR = odds ratio; RCT = randomized controlled trial; RIPC = remote ischemic preconditioning; RD = risk difference; RR = risk ratio; RRT = renal replacement therapy; SAEs = postoperative serious adverse events; sCr = serum creatinine; SR = systematic review; SUCRA = surface under cumulative ranking; TIVA = total intravenous anesthesia; TSA = trial sequential analysis; UO = urine output.

**Table 2:** Consolidated Strength of Recommendations by Intervention.

Intervention	Guideline Recommendation (Class/Level; Source)	Practice Statement in This Review
Goal-directed perfusion	Class I, Level A [54]	Maintain DO_2_ index ≥ 280 mL/min/m^2^ during CPB
Amino acid infusion	Class IIa, Level B [54]	Standard renoprotective strategy
Preoperative anemia correction	No specific class	Correct preoperative anemia whenever feasible
Transfusion strategy (liberal vs. restrictive)	Transfusion during CPB only at Hct < 18%: Class I, Level C or 18–24% with evidence of inadequate oxygenation: Class IIb, Level B [54]	Restrictive strategy preferred
Biomarker-guided KDIGO bundle	1B [53]	Adopt in patients at high biomarker-defined risk
Remote ischemic preconditioning	Class IIa Level A with volatile anesthesia [54]	May be considered under volatile agents
Pulsatile flow during CPB	Class IIa, Level B [54]	May be considered in patients at elevated renal risk
Minimally invasive extracorporeal circulation	Class IIa, Level B [54]	Recommended to reduce blood loss and AF, not for AKI prevention
Dexmedetomidine	Not included in current guidelines	Promising adjunct; no firm recommendation
Intraoperative MAP targets	Class I, Level A to maintain MAP 50–80 mmHg [54]	MAP > 80 mmHg with vasopressors during CPB not recommended for renal protection
Volatile anesthesia vs. TIVA	Class IIb, Level B for volatile maintenance during CPB [54]	Either agent acceptable
N-acetylcysteine	Class IIb, Level B in pre-existing CKD [54]	May be considered in patients with CKD
Hemoadsorption (oXiris)	Class III, Level B in elective surgery; Class IIb, Level B in infective endocarditis [54]	May be considered in infective endocarditis; not in elective cardiac surgery
Natriuretic peptides	2C against use for AKI prevention [55]	Should be considered to reduce the risk of AKI (moderate) and RRT (high certainty)

Class and Level of Evidence are those assigned by the cited clinical practice guidelines and are not recommendations issued by the authors. The practice statements in the final column correspond to the concluding statements of each subsection of Section 3. Abbreviations: as defined in Table 1.

## Data Availability

No new data were created or analyzed in this study.

## Supplementary Materials

The following supporting information can be downloaded at: https://www.mdpi.com/article/10.3390/jcm15176532/s1, Table S1: Additional studies reporting AKI as an outcome in CS-AKI prevention.
