# Supplementary material for: Update on Perioperative Prevention of Cardiac Surgery-Associated Acute Kidney Injury
Source: J Clin Med. 2026 Aug 24;15(17):6532. doi: 10.3390/jcm15176532 (PMC13566849; doi:10.3390/jcm15176532)
Supplement: Supplementary file 1 [file jcm-15-06532-s001.zip › jcm-4473656-supplementary.pdf]

Table S1. Additional Studies Reporting AKI as an Outcome in CS-AKI Prevention

Trial / Author (Year)	n	Design	Country	Intervention vs. Comparator	AKI Endpoint	Key CS-AKI Result
**Amino Acid Infusion**						
**Baiardo Redaelli 2025 [61]**	812	Preplanned secondary analysis of PROTECTION	International	AA vs Placebo (CKD subgroup)	KDIGO creatinine criteria; RRT	**POSITIVE — AKI reduction: 43.1% vs 50.3% RR 0.86 (CI 95% 0.74–0.99); stage 3 AKI: 2.7% vs 5.6% RR 0.48 (CI 95% 0.24–0.98). RRT was numerically lower but not significant**
**Belletti 2025 [62]**	232	Post-hoc subgroup analysis of PROTECTION	International	AA vs. placebo (tMCS subgroup)	KDIGO creatinine criteria	**POSITIVE — AKI reduction: 44.6% vs 60.8% RR 0.73 (CI 95% 0.57–0.94); stage 3 AKI and RRT were not significantly reduced**
**Biomarker-Guided KDIGO Care Bundle**						
**PrevAKI-MC — Zarbock 2021 [71]**	278	RCT	Europe	KDIGO bundle vs. standard care	KDIGO Stage 2–3 AKI within 72h	**POSITIVE — Severe AKI reduction: 14.0% vs. 23.9% OR 0.52 (CI 95% 0.28–0.96)**
**Preoperative Anemia Management**						
**De Santo 2009 [66]**	1,047	Cohort	Italy	Preoperative anemia vs. no anemia (on-pump CABG)	RIFLE	**RISK FACTOR — Preoperative anemia independently predicts AKI: OR 2.06 (CI 95% 1.14–3.70); renal risk operates partly through transfusion exposure**
**Hogervorst 2014 [67]**	11,508	Cohort	Netherlands	≥50% intraoperative Hb decline vs. lesser decline	Composite death/stroke/MI/renal failure	**RISK FACTOR — Relative Hb decline predicts adverse outcomes even when nadir Hb remains above 7 g/dL: aOR 1.27 (CI 95% 1.14–1.41)**

**Khan 2014 [69]**	1,210	Cohort	USA/International	Intraoperative transfusion volume vs. no transfusion	Urinary AKI biomarkers	**RISK FACTOR — Dose-dependent tubular injury with transfusion: ≥4 units associated with 2.1× higher urinary NGAL**
**Transfusion Strategy (Restrictive vs. Liberal)**						
**TRACS — Hajjar 2010 [70]**	502	RCT	Brazil	Restrictive (Hct ≥24%) vs. liberal (Hct ≥30%)	Composite endpoint: including RRT	**NEUTRAL — Restrictive strategy safe but does not reduce AKI 5% vs 4% (p=0.99)**
**Remote Ischemic Preconditioning**						
**HypnoRenalRIP — Zarbock 2025 [75]**	160	RCT	Germany	RIPC vs. sham × propofol vs. sevoflurane	Δ [TIMP-2]·[IGFBP7] pre-post intervention (surrogate renoprotection biomarker)	**POSITIVE (for anesthetic–RIPC interaction) — Transient elevation of [TIMP-2]·[IGFBP7] induced by RIPC observed only with sevoflurane. Postoperative renal stress ([TIMP-2]·[IGFBP7] elevation) was detected in all groups except sevoflurane+RIPC (p=0.001).**
**Pulsatile Flow During CPB**						
**Nam 2015 [80]**	1,372	MA (9 RCTs)	International	Pulsatile vs. non-pulsatile CPB	CrCl, sCr, NGAL, ARI incidence, ARF incidence	**POSITIVE — Effective pulsatile flow reduced ARI and improved CrCl/sCr, but showed no significant effect on ARF or NGAL; benefits were limited to studies with confirmed effective pulsatility. Methodological limitations limit interpretation**
**Minimally Invasive Extracorporeal Circulation**						

**Cheng 2021 [84]**	4,350	MA (42 RCTs)	International	MiECC vs. CECC	Mostly AKIN Stage 1/ composite endpoint	**NEUTRAL — No isolated renal benefit: renal failure not significantly reduced as independent endpoint. Composite significantly reduced: OR 0.64 (CI 95% 0.50–0.81)**
**Intraoperative MAP Targets**						
**Azau 2014 [93]**	292	RCT	France	MAP 75–85 vs. 50–60 mmHg (norepinephrine)	≥30% sCr rise (primary); RIFLE categories (secondary)	**NEUTRAL — Higher MAP with vasopressors provides no renal benefit: AKI 17% vs 16.7% OR 1.03 (CI 95% 0.56–1.91)**
**Kandler 2019 [92]**	90	RCT	Denmark	MAP >60 vs. 47 mmHg during CPB	GFR (Cr-EDTA clearance) at 4 months; RIFLE criteria; urinary NGAL	**NEGATIVE — Higher MAP does not preserve GFR; no benefit at 4-month follow-up. no AKI reduction by RIFLE (46% vs. 38%, p=0.447); NGAL comparable**
**Volatile Anesthesia vs TIVA**						
**Deng 2024 [97]**	3,083	RCT	China	Volatile vs. TIVA	Composite including AKI and acute kidney dysfunction	**NEUTRAL — No AKI benefit: OR 0.97 (CI 95% 0.64–1.47); no benefit in composite primary outcome RR 1.03 (CI 95% 0.88–1.21)**
**N-Acetylcysteine**						
**He 2018 [98]**	678	MA (5 RCTs)	International	NAC vs placebo in CKD/pre-existing renal insufficiency	sCr rise within 48–72 h	**POSITIVE (SIGNAL) — NAC reduced AKI overall RR 0.82, (CI 95% 0.69–0.99), especially with IV NAC RR 0.77 (CI 95% 0.63–0.94), but did not reduce RRT.**
**Extracorporeal Blood Purification**						
**REMOVE — Diab 2022 [103]**	288	RCT	International	CytoSorb vs. standard care	SOFA score; organ dysfunction	**NEGATIVE — CytoSorb provides no**

				(infective endocarditis)		**organ protection in IE: ΔSOFA 1.79 vs. 1.93 (p=0.677); no benefit in 30-day mortality or any secondary endpoint**
**Dexmedetomidine**						
**Soh 2020 [90]**	108	RCT	South Korea	Dexmedetomidine 0.4 µg/kg/h vs placebo	KDIGO	**POSITIVE — Significant AKI reduction in aortic surgery: 13% vs. 31% OR 0.32 (CI 95% 0.12–0.86)**
**Zhao 2024 [88]**	2,907	MA (15 RCTs)	International	Dexmedetomidine vs. placebo	AKI incidence	**POSITIVE — AKI reduction confirmed in second independent meta-analysis: OR 0.66 (CI 95% 0.48–0.91; p=0.01)**
**Li 2025 [87]**	2,744	MA (14 RCTs)	International	Dexmedetomidine vs placebo	RIFLE, AKIN, KDIGO	**POSITIVE — Consistent AKI reduction with parallel creatinine benefit: RR 0.54 (CI 95% 0.41–0.70)**
**Levosimendan**						
**LICORN — Cholley 2017 [102]**	336	RCT	France	Levosimendan vs. placebo	LCOS composite including prolonged catecholamine infusion, mechanical assist device, or RRT during ICU stay; RRT also assessed as a secondary endpoint	**NEGATIVE — No reduction in LCOS composite (52% vs 61%; RD −7%, CI 95% −17 to 3; p=0.15); RRT not reduced (9% vs 6%; p=0.16); no 180-day mortality benefit.**
**Elbadawi 2018 [101]**	2,273	MA (16 RCTs)	International	Levosimendan vs placebo or active control	AKI as secondary outcome (various definitions); dialysis	**POSITIVE — AKI 41% relative reduction (RR 0.59, CI 95% 0.38–0.92). No significant reduction in postoperative dialysis was observed (RR 0.71, CI 95% 0.43–1.16)**
**Natriuretic Peptides (ANP)**						
**NU-HIT — Sezai 2009 [104]**	504	RCT	Japan	Carperitide vs. placebo	Postoperative renal dysfunction: sCr	**POSITIVE — hANP preserved creatinine**

					increase ≥0.3 mg/dL, Cr >2.0 mg/dL, creatinine clearance, and hemodialysis	**clearance and reduced postoperative renal dysfunction: sCr increase ≥0.3 mg/dL occurred in 40 vs 92 patients (p<0.0001), and Cr >2.0 mg/dL in 1 vs 8 patients (p=0.0374). Hemodialysis was numerically lower but not significant: 0 vs 4 patients (p=0.123).**
**Yamada 2019 [105]**	1,471	SR/MA (18 RCTs)	International	hANP vs. placebo	AKI no specific criteria; RRT	**POSITIVE — hANP reduced new AKI in prevention trials RR 0.51 (CI 95% 0.36–0.72) and RRT requirement RR 0.17 (CI 95% 0.04–0.64)**

Abbreviations: as defined in Table 1.
